# Prediction of phenolic compounds and glucose content from dilute inorganic acid pretreatment of lignocellulosic biomass using artificial neural network modeling

**DOI:** 10.1186/s40643-021-00488-x

**Published:** 2021-12-19

**Authors:** Hongzhen Luo, Lei Gao, Zheng Liu, Yongjiang Shi, Fang Xie, Muhammad Bilal, Rongling Yang, Mohammad J. Taherzadeh

**Affiliations:** 1 School of Life Science and Food Engineering, Huaiyin Institute of Technology, 1 Meicheng East Road, Huaian, 223003 China; 2 Jiangsu Provincial Engineering Laboratory for Biomass Conversion and Process Integration, Huaiyin Institute of Technology, Huaian, 223003 China; 3 Faculty of Applied Technology, Huaiyin Institute of Technology, Huaian, 223003 China; 4 Swedish Centre for Resource Recovery, University of Borås, 50190 Borås, Sweden

**Keywords:** Lignocellulosic biomass, Dilute acid pretreatment, Enzymatic hydrolysis, Phenolic compounds, Artificial neural network, Modeling

## Abstract

Dilute inorganic acids hydrolysis is one of the most promising pretreatment strategies with high recovery of fermentable sugars and low cost for sustainable production of biofuels and chemicals from lignocellulosic biomass. The diverse phenolics derived from lignin degradation during pretreatment are the main inhibitors for enzymatic hydrolysis and fermentation. However, the content features of derived phenolics and produced glucose under different conditions are still unclear due to the highly non-linear characteristic of biomass pretreatment. Here, an artificial neural network (ANN) model was developed for simultaneous prediction of the derived phenolic contents (*C*_Phe_) and glucose yield (*C*_Glc_) in corn stover hydrolysate before microbial fermentation by integrating dilute acid pretreatment and enzymatic hydrolysis. Six processing parameters including inorganic acid concentration (*C*_IA_), pretreatment temperature (*T*), residence time (*t*), solid-to-liquid ratio (*R*_SL_), kinds of inorganic acids (*k*_IA_), and enzyme loading dosage (*E*) were used as input variables. The *C*_Phe_ and *C*_Glc_ were set as the two output variables. An optimized topology structure of 6–12-2 in the ANN model was determined by comparing root means square errors, which has a better prediction efficiency for *C*_Phe_ (*R*^2^ = 0.904) and *C*_Glc_ (*R*^2^ = 0.906). Additionally, the relative importance of six input variables on *C*_Phe_ and *C*_Glc_ was firstly calculated by the Garson equation with net weight matrixes. The results indicated that *C*_IA_ had strong effects (22%-23%) on *C*_Phe_ or *C*_Glc_, then followed by *E* and *T*. In conclusion, the findings provide new insights into the sustainable development and inverse optimization of biorefinery process from ANN modeling perspectives.

**Graphical Abstract:**

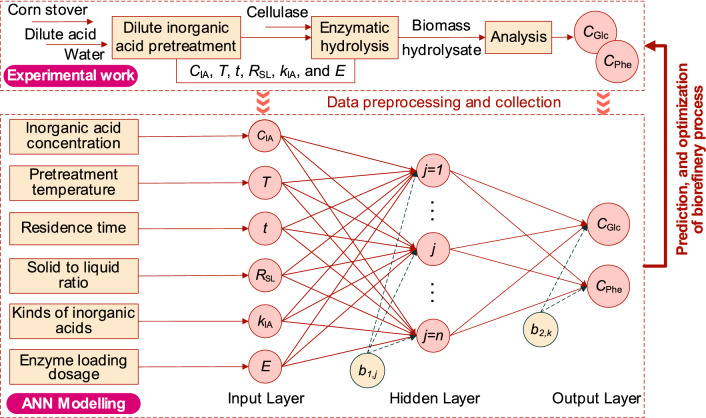

**Supplementary Information:**

The online version contains supplementary material available at 10.1186/s40643-021-00488-x.

## Introduction

Nowadays, the concerns over climate change, especially the increasing greenhouse gases (GHGs) emissions, have necessitated a rethinking of traditional methods for fuels production (Field et al. 2020; Solarte-Toro et al. 2019). According to the statistics, about 25% of the total GHGs emission was contributed by transportation (Keasling et al. 2021). Reducing GHGs emissions and enhancing carbon capture/sequestration (CCS) are global concerns (Ishaq et al. 2021). To this end, one avenue is to produce advanced transportation fuels from atmospheric carbon resources (mainly CO_2_) or renewable biomass grown by fixing CO_2_ to partially replace fossil resources (Liu et al. 2020).

In the past decades, utilization of lignocellulosic biomass from non-food crops for production of fuels and fine chemicals has gained much attention (Luo et al. 2021a; Rajan et al. 2020) because their net carbon footprint is neutral. Lignin (15–30 wt%), cellulose (30–50 wt%), and hemicellulose (20–35 wt%) are three main components in lignocellulose (Schutyser et al. 2018). The structure of lignocellulose is complex since cellulose and hemicellulose are enwrapped by lignin, and hemicellulose is also interlaced with cellulose fibers (Liu et al. 2021), resulting in the biomass recalcitrance and low enzymatic efficiency for hydrolysis. Depolymerization of lignocellulose to obtain fermentable sugars (mainly glucose) is the key for production of fuels (Luo et al. 2018). Thus, decreasing biomass recalcitrance and structural complexity via efficient pretreatments is generally required (Liu et al. 2021).

To disrupt the close inter-component association between cellulose, hemicellulose, and lignin, various pretreatment strategies including acid-, alkaline-, ionic liquid-, and organic solvent-based methods have been developed (Hijosa-Valsero et al. 2017; Jönsson and Martín 2016; Xia et al. 2021). Among those pretreatment strategies, dilute inorganic acids hydrolysis is one of the most promising methods with high recovery of fermentable sugars and low cost (Jönsson and Martín 2016), which is beneficial for the production of biofuels and chemicals. Nevertheless, during acid pretreatment of biomass, hemicellulose and lignin are partially solubilized, which result in the degradation of these fragments under an acidic environment (Zhang et al. 2021). Generally, three kinds of inhibitors, including weak organic acids (acetic acid, formic acid, etc.), furan derivatives, and phenolics (phenolic acids, and phenolic aldehydes) are derived during pretreatment, which affect enzymatic and fermentation efficiency (Chen et al. 2020; Yao et al. 2021). Especially, the phenolic compounds derived from lignin degradation during pretreatment characterized by complex structure, diversity, low water solubility, and low hydrophobicity were reported as the main limiting factor to the industrial biofuel production (Gu et al. 2019).

To counteract the toxic effect of phenolics on the enzymatic and fermentation process, detoxification of lignocellulosic hydrolysates and slurries with overliming, activated carbon, or water washing is widely implemented (Sivagurunathan et al. 2017). However, fermentable sugars were also partially removed, and the treatment of generated wastewater would further deteriorate techno-economic performance. Additionally, the construction of robust strains by elucidating the response mechanism to phenolics could also weaken the inhibitory effect (Kumar et al. 2020; Luo et al. 2021b; Luo et al. 2020). For example, Jiménez-Bonilla et al. (2020) reported that overexpressing efflux pump gene *srpB* from *Pseudomonas putida* is beneficial to improve the tolerance of *Clostridium saccharoperbutylacetonicum* to 1.2 g/L ferulic acid (Jiménez-Bonilla et al. 2020). Although systems metabolic engineering and adaptive evolution strategies could improve the robustness of microbes under biomass-derived inhibitors stress, the derived concentration feature of inhibitors in biomass hydrolysate under pretreatment process should be firstly considered.

Optimization of pretreatment conditions by evaluating glucose hydrolysis efficiency and derived phenolics was carried out using different lignocellulose such as rice straw (Lee et al. 2012), sugarcane bagasse (Lv et al. 2017), etc. The content of lignin-derived phenolics in hydrolysate before fermentation is mainly attributed to pretreatment conditions, such as biomass species, pretreatment temperature, reaction time, solid-to-liquid ratio, etc. (Bhatia et al. 2020; Jönsson and Martín 2016), and also changed during enzymatic hydrolysis (Yao et al. 2021). Implementing numerous experiments for pretreatment of biomass could achieve a sub-optimal result, it would unavoidably increase the operation complexity and time-consuming. Loading of high-cost cellulase also largely improves the total biorefinery cost. Furthermore, the relationship between pretreatment, enzymatic conditions, and derived features of phenolics content was still unclear. Hence, it remains challenging to systematically analyze the effects on derived phenolics and glucose yield concerning both biomass characteristics, pretreatment, and enzymatic conditions.

Development of bioprocess modeling for the non-linear lignocellulosic bioprocessing is an efficient strategy enabling the success of biorefinery and bio-based circular economy (Unrean 2016). Recently, artificial intelligence (AI) technology, mainly machine learning (ML) algorithms, is competent for predicting/confirming relative importance between input and output variables (Li et al. 2021). The effectiveness of ML methods for predicting pyrolytic gas yield and compositions was verified (Tang et al. 2021), which could benefit to better understand biomass pyrolysis and syngas upgrading. An artificial neural network (ANN) model with a multilayer architecture (3-15-1) was optimized and predicted the biogas production curve from cattle under mesophilic and thermophilic conditions (Ghatak and Ghatak 2018). An ANN model was built to predict sugar yields of pretreated rice straw during hydrolysis by considering three factors of biomass loadings, particle size, and reaction time (Vani et al. 2015). Recently, Moodley et al. (2019) found that sugar yield from sugarcane leaf waste was sensitive to the alkali and salt concentrations by establishing two ANN tools in inorganic salt pretreatment process (Moodley et al. 2019). The above reports clearly show that ANN can be trained with experimental data to generate efficient models of non-linear multivariate processes. To the best of our knowledge, prediction of lignin-derived phenolics content and glucose concentration from inorganic acid pretreatment of biomass with advanced modeling technology was not reported thus far.

Focusing on above-mentioned issues, in this study, we aim to develop an ANN model for elucidating the derived feature of phenolics from corn stover by integrative investigating typical three inorganic acids (HCl, H_2_SO_4_, and H_3_PO_4_) pretreatment and enzymatic hydrolysis processes. Furthermore, the relative importance of pretreatment conditions (i.e*.*, input variables) on phenolic and glucose concentrations (i.e*.*, output variables) was also first elucidated by considering the neural net weights in the developed ANN model. The results would provide new insights into the biorefinery process for biofuels production.

## Materials and methods

### Materials and chemicals

The corn stover was collected from Lianyungang City, China. It was firstly cut and sieved to a particle size of ~ 0.4 mm. The fine corn stover was then dried in an oven (GZX-9140MBE, Shanghai Boxun Medical Biological Instrument Corp., China) at 60 °C for 12 h to remove the moisture and stored in plastic bags at 4 °C. Three kinds of inorganic acids, including dibasic acid (hydrochloric acid, 37 wt%), binary acid (sulfuric acid, 98 wt%), and ternary acid (phosphoric acid, 85 wt%) were used as pretreatment agents for corn stover depolymerization. The inorganic acids were purchased from Sinopharm Chemical Reagent Co., Ltd. All of the chemicals were used as received without other specified purification. A commercial cellulase Cellic CTec2 (enzyme blend, SAE0020-50 mL solution) was obtained from Sigma-Aldrich (St. Louis, MO, USA), and used for hydrolyzing the pretreated corn stover to obtain glucose.

### Dilute inorganic acid pretreatment and enzymatic hydrolysis

To systematically investigate the effects of pretreatment conditions on the derived phenolic compounds and glucose hydrolysis yield, six key parameters including inorganic acid concentration (*C*_IA_), pretreatment temperature (*T*), residence time (*t*), solid-to-liquid ratio (*R*_SL_), kinds of inorganic acids (*k*_IA_), and enzyme loading dosage (*E*) were considered in this study. The acid pretreatment process of corn stover was carried out in a 250-mL vertical reactor (TGYF-B, Gongyi Yuhua Instrument Co., Ltd., China) with an electrically magnetic stirrer and a temperature controller. Firstly, the dried corn stover, inorganic acid, and 150 mL water were added into the reactor simultaneously. The experimental parameter ranges for corn stover pretreatment, and enzymatic hydrolysis were carefully designed and also summarized in Table 1, containing the raw data for training, validation, and testing the following ANN model. After acid hydrolysis, the pH of the pretreated mixture was regulated to 5.0 by 8 mol/L NaOH solution. The overall effects of biomass-derived phenolic compounds on enzymatic hydrolysis were also considered due to the interaction of phenolics with cellulase. Thus, the pretreated mixture was not filtrated, and directly used for enzymatic hydrolysis by adding cellulase with 10–20 FPU/g corn stover (Table 1). The reaction was conducted at 50 °C in a water bath at 150 rpm. After 72 h enzymatic hydrolysis, the pretreated mixture was firstly boiled at 95–100 °C for 5 min to terminate the reaction. Then, the liquid fraction of the mixture (i.e*.,* hydrolysate) was separated by vacuum filtration for determining phenolics and glucose contents.

**Table 1:** The design of operating conditions to perform the dilute acid pretreatment of lignocellulosic biomass for the development of ANN model

Lignocellulose	Kinds of acids (*k*_IA_)^a^	Acid concentration (*C*_IA_, mol/L)	Pretreatment temperature (*T*, ℃)	Residence time (*t*, min)	Solid to liquid ratio (*R*_SL_)	Enzyme loading dosage (*E*, FPU/g)
Corn stover	Hydrochloric acid (HCl)	0.05–0.6	120–200	20–60	10–15%	10–20
	Sulfuric acid (H_2_SO_4_)	0.05–0.6	120–200	20–60	10–15%	10–20
	Phosphoric acid (H_3_PO_4_)	0.05–0.6	120–200	20–60	10–15%	10–20

The pretreatment experiments with specified conditions were all carried out with three replicates. The raw data used for ANN development were the means and the detailed means ± SD values are shown in Table S1

Three kinds of inorganic acid including dibasic acid (HCl), binary acid (H_2_SO_4_), and ternary acid (H_3_PO_4_) were used

^a^The *k* value for HCl, H_2_SO_4_ and H_3_PO_4_ was set as 1, 2, and 3, respectively, which was used as one input parameter for the development of ANN model

### Development of ANN model

#### Selection of input variables and experimental data

Different kinds of biomass (corn stover, rice straw, switchgrass, sugarcane straw, etc.) have different ratios of cellulose, hemicellulose, and lignin, which result in various features of the derived phenolics and glucose yield even under the same pretreatment and enzymatic hydrolysis conditions (Pratto et al. 2020; Solarte-Toro et al. 2019). Among these lignocellulosic biomasses, corn stover is the largest crop residue in China (Yang et al. 2020b); thus, it was selected as the model biomass for the pretreatment experiments in this study.

The formation of the biomass-derived phenolic compounds content and glucose hydrolysis yield in hydrolysate is a complex and non-linear process, which is difficult to directly predict the derived features with traditional constructive mathematical models. Thus, we tried to use ANN modeling to predict derived phenolic compounds content and glucose yield after pretreatment and enzymatic hydrolysis processes. As described in above section, six key parameters of *C*_IA_, *T*, *t*, *R*_SL_, *k*_IA_, and *E* (Table 1) were considered as the input variables for the development of ANN model. The output variables were glucose concentration (*C*_Glc_) and phenolic content (*C*_Phe_) in biomass hydrolysate after 72 h enzymatic hydrolysis. To better perform the ANN model, 77 runs experiments were carried out according to the design of input variables shown in Table 1.

#### Data preprocessing, and the topology of ANN model

**Fig. 1:**
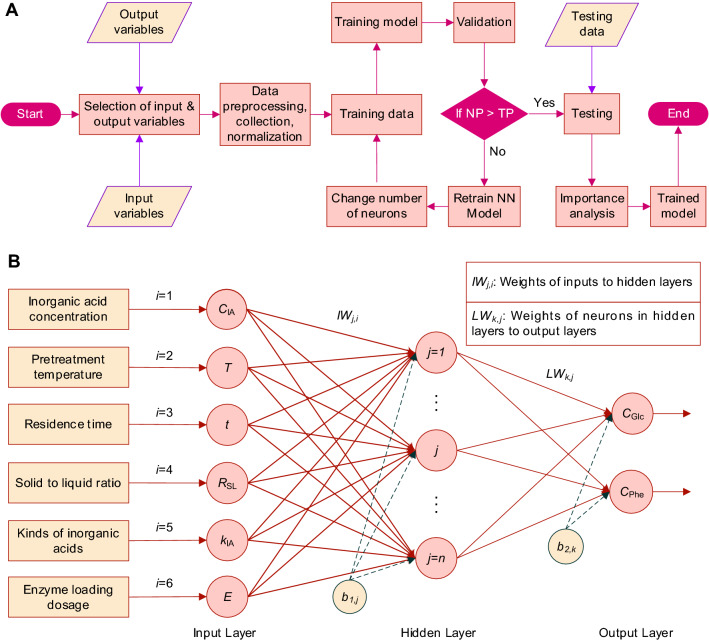
The flow diagram of the development of ANN model (**A**) and the topology structure of ANN model (**B**) to predict the glucose concentration (*C*_Glc_) and total phenolic content (*C*_Phe_) in biomass hydrolysate after dilute inorganic acid pretreatment and 72 h enzymatic processes. **A** NP, network performance; TP, target performance of network; NN, neural network. **B** *b*_1,j_, the bias of inputs (*j* = 1, 2, 3, …, *n*); *b*_2,k_, the bias of output layer (*k* = 1, 2)

Figure 1A shows the step-by-step scheme of ANN model development to predict *C*_Phe_ and *C*_Glc_ from corn stover under different operation conditions. The ANN model was developed by using Matlab R2019a (The MathWorks, Inc., USA). It has one multiple layer neural network with interconnected neurons arranged in three layers of input, hidden, and output layers (Fig. 1B). The ANN model proposed in present study consists in: (1) two parameters of *C*_Glc_ and *C*_Phe_ were considered the output variables; (2) six variables of *C*_IA_, *T*, *t*, *R*_SL_, *k*_IA_, and *E* in one input layer were fully connected to the hidden layer; (3) one hidden layer had *n* neurons; and (4) bias of *b*_1,j_ (the bias of inputs, *j* = 1, 2, 3, …, *n*) and *b*_2,k_ (the bias of output layer, *k* = 1, 2) were used for training the network.

Firstly, the whole datasets were split as training, validation, and testing groups with a ratio of 75:15:10. The input dataset is normalized in the range of [− 1 1] before training the network to obtain an accurate model. The inbuilt ‘mapminmax’ function is used for the normalization of experimental data, which is equivalent to Eq. (1):

1 $$V^{\prime} = \frac{{V - V_{\min } }}{{V_{\max } - V_{\min } }}\left( {V^{\prime}_{\max } - V^{\prime}_{\min } } \right) + V^{\prime}_{\min } ,$$

where *V*′, *V*, *V*_max_, *V*_min_, *V*′_max_, and *V*′_min_ represented the new value, the original value, the original maximum limit, original minimum limit, the new maximum limit (i.e., 1), and the new minimum limit (i.e., − 1), respectively.

The ANN model was trained by the Adam optimizer, with a learning rate of 0.001, and training batches of size 2. The neurons (*n*) in the hidden layer were determined by an empirical equation Eq. (2) (Yang et al. 2020a). The root means square error (RMSE) obtained from different neurons and iterations were used to evaluate the accuracy of model predictions, which was calculated by Eq. (3). Based on RMSE results, an optimized ANN model with better performance was developed to predict *C*_Glc_ and *C*_Phe_ (Fig. 1A):

2 $$n = \sqrt {i + k} + \alpha ,$$

3 $${\text{RMSE}} = \sqrt {\frac{1}{m}\sum\limits_{h = 1}^{m} {\left( {y_{{{\text{pre}}}}^{(h)} - \hat{y}_{\exp }^{(h)} } \right)} } ,$$

where *n* is the number of neurons in hidden layer; *i* is the number of input variables; *k* is the number of output variables; *α* is a constant range of 1–10; *y*_pre_^(h)^ is the predicted output value of *C*_Phe_; ${\hat{\text{y}}}$_exp_^(h)^ is the experimental value of the output variable of *C*_Phe_; and *m* is the number of the samples for training, validation, or testing of ANN models.

#### Analysis of relative importance of input variables

The parameters of *IW*_j,i_, *LW*_k,j_, *b*_1,j_, and *b*_2,k_ in the developed ANN model could be used to simulate the output variables (*C*_Glc_, *C*_Phe_). In addition, to evaluate the relative importance of the input variables on the two output variables, the process was based on the neural net weight matrixes (*IW*_j,i_, and *LW*_k,j_, Fig. 1B) and Garson equation (Puig-Arnavat et al. 2013; Sunphorka et al. 2017). Garson equation was based on the partitioning of connection weights in the network. The numerator presents the total of absolute products of weights for each input (*i* = 1, 2…6) while the denominator represents the total of the absolute values of all weights feeding into the hidden layer (*j* = 1, 2…*n*). The Garson equation is presented in Eq. (4) for adapting the ANN topology:

4 $${I_i} = \frac{{\sum\limits_{j = 1}^n {\left( {\left( {\left| {I{W_{j,i}}} \right| \div \sum\limits_{i = 1}^{i = 6} {\left| {I{W_{j,i}}} \right|} } \right) \times \left| {L{W_{k,j}}} \right|} \right)} }}{{\sum\limits_{i = 1}^{i = 6} {\left\{ {\sum\limits_{j = 1}^n {\left( {\left( {\left| {I{W_{j,i}}} \right| \div \sum\limits_{i = 1}^{i = 6} {\left| {I{W_{j,i}}} \right|} } \right) \times \left| {L{W_{k,j}}} \right|} \right)} } \right\}} }} \times 100\% ,$$

where *I*_i_ is the relative importance of the *i*th input variable on output variables of *C*_Glc_, and *C*_Phe_; *IW*_j,i_ is the neural net weight to *j*th neuron of the hidden layer from *i*th input variable; and *LW*_k,j_ is the weight to *k*th output variable from *j*th neuron of the hidden layer, respectively.

### Analytical methods

The glucose concentration in corn stover hydrolysate (*C*_Glc_) was determined by a biosensor analyzer (S-10, Sieman Technology, China) (Luo et al. 2019). Determination of total phenolic content (*C*_Phe_) in the hydrolysate was based on the Folin–Ciocalteu assay with gallic acid as the standard (Xu et al. 2021) with some modifications. Briefly, 0.4 mL sample was mixed with 2.6 mL water and 0.5 mL Folin–Ciocalteu reagent (1.0 mol/L, Sinopharm Chemical Reagent Co., Ltd., Shanghai). After 5 min, 5.0 mL water and 1.5 mL Na_2_CO_3_ solution (20%, w/v) was added simultaneously. Then, the mixture oscillated under a dark environment at 40 °C for 1 h. Finally, the absorbance of the reaction mixture was analyzed at 760 nm by a UV–Vis spectrophotometer (UV-2100, Unico Instrument Co., Ltd., China). As a result, the *C*_Phe_ (g/L) was calculated by Eq. (5):

5 $$C_{{{\text{Phe}}}} = \frac{{a \times A_{760} }}{{V_{s} }} \times N,$$

where *a* is the linear coefficient of standard curve; *A*_760_ is the absorbance of reaction mixture at 760 nm; *V*_S_ is the volume of reaction mixture; and *N* is the dilution ratio, respectively.

### Statistical analysis

The experimental data of *C*_Phe_ and *C*_Glc_ for development of ANN model are represented as the mean ± standard deviation (SD) of three independent experiments. Significant differences were confirmed with a two-tailed Student’s *t*-test aided by Microsoft Excel 2016.

## Results

### Effects of inorganic acid pretreatment/enzymatic hydrolysis on the content of derived phenolics and glucose

**Fig. 2:**
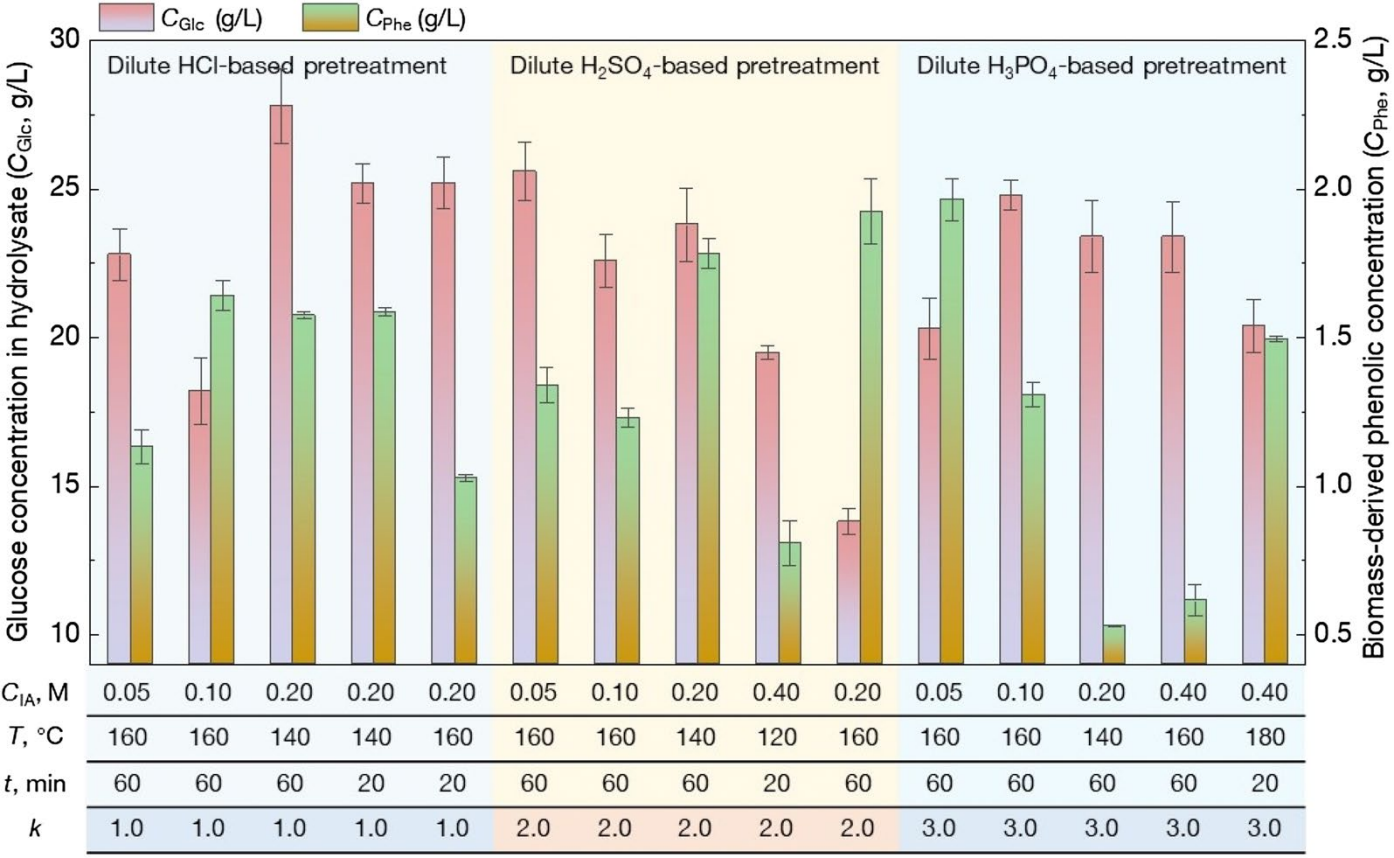
Effects of dilute inorganic acid pretreatment of corn stover on the glucose concentration (*C*_Glc_) and derived phenolic concentration (*C*_Phe_) in hydrolysate after 72 h enzymatic hydrolysis. The typical 15 batch experiments results were selected from Table S1 with same values of the ratio of solid to liquid (*R*_SL_, 0.10) and cellulase loading dosage (*E*, 20 FPU/g corn stover)

For efficient production of biofuels and fine chemicals from lignocellulosic biomass via microbial fermentation, an optimized pretreatment process featured with a high glucose yield from feedstock and a low derived concentration of inhibitors is crucial (Liu et al. 2021). Inorganic acid-based pretreatment is applied to efficiently solubilize hemicellulose from lignocellulose and it also improves the cellulose digestibility (Jönsson and Martín 2016; Zabed et al. 2016). Therefore, dilute inorganic acids hydrolysis is widely used in biorefinery process. In this study, focusing on dilute inorganic acid pretreatment of corn stover, the overall effects of pretreatment conditions on phenolics and glucose concentration after 72 h enzymatic hydrolysis were studied, and the results are shown in Fig. 2. Three typical inorganic acids, including HCl (dibasic acid), H_2_SO_4_ (binary acid), and H_3_PO_4_ (ternary acid) were used as the pretreatment reagent. It should be noted, in these cases, the solid-to-liquid ratio (*R*_SL_), and cellulase loading dosage (*E*) was kept at 10% and 20 FPU/g corn stover, respectively.

When pretreating corn stover with 0.05 mol/L of HCl under 160 °C for 60 min, the derived phenolic compounds concentration in hydrolysate (*C*_Phe_) was 1.13 g/L, and glucose content (*C*_Glc_) reached 22.8 g/L. Under the same condition of H_2_SO_4_ pretreatment, *C*_Glc_ were increased by 12.3% (25.6 g/L), and *C*_Phe_ was also elevated to 1.34 g/L with significant differences (*p* < 0.05) to the HCl pretreatment process. Whereas, in the case of 0.05 mol/L H_3_PO_4_ pretreatment with 160 °C for 60 min, the glucose yield was only 20.3 g/L with a higher level of *C*_Phe_ (1.97 g/L, Fig. 2). The results indicated that cellulase might tolerate a low concentration of *C*_Phe_. For the HCl pretreatment of corn stover, *C*_Phe_ was increased to 1.64 g/L when elevating acid concentration from 0.05 to 0.1 mol/L, but the phenomenon was not found in H_2_SO_4_ and H_3_PO_4_ pretreatment processes. In addition, the effects of different *R*_SL_ and *E* on *C*_Glc_ and *C*_Phe_ were also studied and the detailed experimental data are also shown in Additional file 1: Table S1. Since glucose and derived phenolic contents are mainly attributed to complex pretreatment conditions and enzymatic process with multivariate non-linear features (Huang et al. 2007), it is still challenging to speculate the changing patterns of *C*_Glc_ and *C*_Phe_ by only implementing numerous experiments. Hence, it is necessary to explore advanced methods for elucidating the relative importance of operational conditions on *C*_Glc_ or *C*_Phe_ for further optimizing the lignocellulose pretreatment process.

### Optimization and determination of key parameters in ANN model

**Fig. 3:**
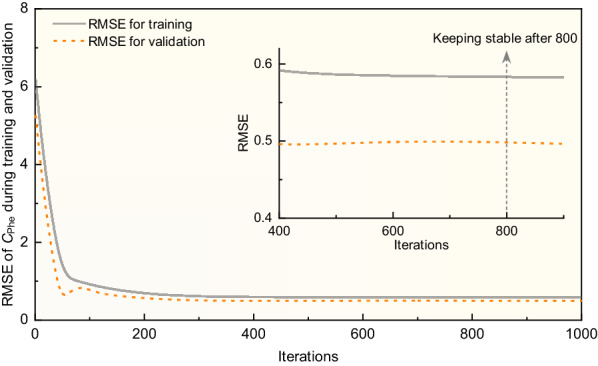
The changing patterns of RMSE (*C*_Phe_) during training and validation with 1000 iterations for the development of ANN models. The subplot is the enlarged visualization of RMSE under 400–900 iterations

As shown in Fig. 1B, the multilayer ANN model for the prediction of *C*_Glc_ and *C*_Phe_ consisted of 6 neurons in one input layer, one hidden layer, and one output layer. Since the number of neurons in the hidden layer is a key parameter in ANN model, and trial–error approach was applied to ensure a relatively fast and good convergence of RMSE (Puig-Arnavat et al. 2013). The obtained results of 11 ANN models under different neuron numbers (*n*) with 500 iterations are exhibited in Table 2. Here, an empirical equation Eq. (2) was used to determine the range of neuron numbers (from 3 to 13) in the hidden layer (Yang et al. 2020a). As shown in Table 2, when the neurons in the hidden layer was 3, RMSE for *C*_Glc_ reached 7.16 in the training dataset and 7.12 in the validation dataset. For the RMSE of *C*_Phe_, a range of 0.41–0.54 was obtained in the case of 3 neurons. When changing the neurons from 3 to 13 in the hidden layer, RMSE varied due to the different parameters while developing ANN models. By combination analysis of the RMSE for *C*_Glc_ and *C*_Phe_, the network performance of ANN model with 12 neurons was better which showed relative lower values of RMSE (Table 2). In addition, the effects of iterations on RMSE for *C*_Phe_ when training and validation of the ANN models were also investigated. The RMSE changing patterns in Fig. 3 indicated that increasing iterations from 400 to 800 could effectively decrease the RMSE of *C*_Phe_ and then kept stable after 800 iterations. Therefore, an optimized iteration of 800 with the best network structure of 6-12-2 was considered.

**Table 2:** Computational results of RMSE during the training and validation processes with different neurons of hidden layer in ANN models. RMSE is the root means square error calculated by Eq. (3)

Neurons in hidden layer, *n*	RMSE for *C*_Glc_ after 500 iterations		RMSE for *C*_Phe_ after 500 iterations	
	Training dataset	Validation dataset	Training dataset	Validation dataset
*n* = 3	7.16	7.12	0.54	0.41
*n* = 4	9.35	8.51	0.87	0.42
*n* = 5	7.92	6.06	0.56	0.18
*n* = 6	6.71	6.41	1.37	1.05
*n* = 7	6.44	5.47	0.46	0.22
*n* = 8	7.53	5.84	0.46	0.46
*n* = 9	6.41	5.03	0.42	0.40
*n* = 10	6.40	5.20	0.43	0.28
*n* = 11	6.57	4.97	0.53	0.25
*n* = 12	6.38	4.73	0.43	0.24
*n* = 13	6.50	5.46	0.43	0.32

### Training and testing of the ANN model

**Fig. 4:**
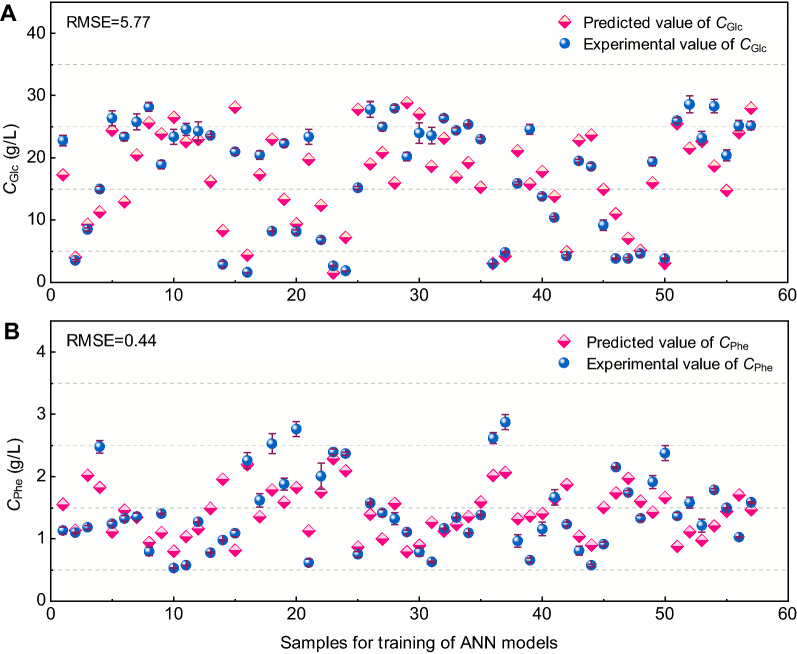
Predicted and experimental values of *C*_Glc_ (**A**) and *C*_Phe_ (**B**) during training ANN model. The experimental values of *C*_Glc_ and *C*_Phe_ were the means ± SD obtained from three replicates. The input values for training ANN model were the means from three experiments, and thus no error bars in the predicted values *C*_Glc_ and *C*_Phe_ in **A** and **B**

The best ANN structure was achieved after numerical experiments using training and validation datasets with a single hidden layer consisted of *n* = 12 neurons (Fig. 1B; Table 2). During the training process with 57 batches dataset (each including 6 input variables and 2 output variables, Additional file 1: Table S1), the predicted values of *C*_Glc_ and *C*_Phe_ are exhibited in Fig. 4. For the predicted values of *C*_Glc_ during training process, the RMSE was 5.77 (Fig. 4A), and the corresponding result for *C*_Phe_ reached a lower value of 0.44 (Fig. 4B). In addition, the optimized weights (*IW*_j,i_, and *LW*_k,j_) and biases (*b*_1,j_, and *b*_2,k_) of the proposed ANN model are listed in Table 3.

**Table 3:** Weights and biases of the hidden and output layers used in the developed ANN model for the prediction of *C*_Glc_ and *C*_Phe_

Node, *j*	Weights and biases of the hidden layer							Weights and biases of the output layer		
	*IW*_j,1_	*IW*_j,2_	*IW*_j,3_	*IW*_j,4_	*IW*_j,5_	*IW*_j,6_	*b*_1,j_	*LW*_1,j_	*LW*_2,j_	*b*_2,k_
1	0.4211	0.5806	0.1236	0.2909	0.1691	0.5445	− 0.0095	0.0448	0.4378	− 0.0381
2	0.3931	0.1473	0.2595	0.4280	0.2980	0.4272	− 0.0393	0.3972	0.4253	− 0.0051
3	0.3056	0.1740	0.3713	0.1522	0.1229	0.1367	− 0.0287	0.0356	0.2299	
4	0.5184	0.0507	0.2491	0.0508	0.3027	0.4435	− 0.0379	0.6842	0.1607	
5	0.3112	0.4807	0.7085	0.2731	0.3305	0.6146	0.0394	0.2159	0.0281	
6	0.1854	0.5232	0.0776	0.4782	0.3439	0.1783	− 0.0306	0.1249	0.4419	
7	0.0370	0.2334	0.0454	0.3650	0.2354	0.4857	0.0372	0.3608	0.0639	
8	0.2985	0.2512	0.3173	0.1907	0.1834	0.5006	0.0361	0.5057	0.4299	
9	0.3070	0.4097	0.0005	0.5172	0.3769	0.2365	− 0.0184	0.0140	0.3469	
10	0.3136	0.2505	0.1438	0.0805	0.0552	0.1178	− 0.0320	0.2159	0.6152	
11	0.6273	0.0770	0.3091	0.3422	0.3044	0.3722	0.0394	0.6076	0.3147	
12	0.5133	0.5409	0.0001	0.4964	0.2515	0.1423	0.0371	0.5611	0.2426	

*j* is the neuron node in the hidden layer (from *j* = 1 to 12). *IW*_j,i_ (*i* = 1, 2, 3, 4, 5, and 6) is the neural net weight to *j*th neuron of the hidden layer from *i*th input variable; and *LW*_k,j_ (*k* = 1 and 2) is the neural net weight to *k*th output variable from *j*th neuron of the hidden layer. *b*_1,j_ is the bias of input variables, and *b*_2,k_ is the bias of output layer

**Fig. 5:**
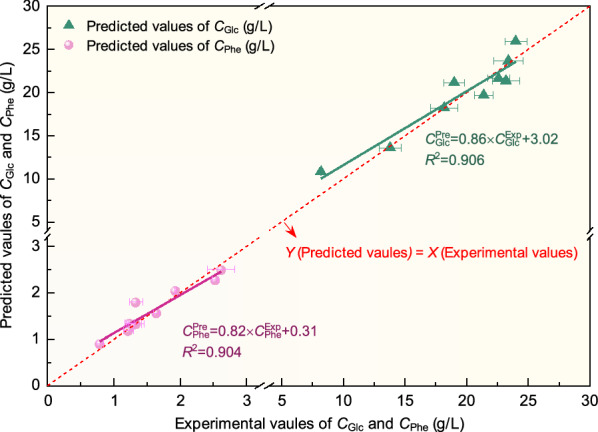
Experimental validation and fitting relationship of *C*_Glc_ and *C*_Phe_ based on the developed ANN model. *C*^Exp^_Glc_, experimental values of *C*_Glc_; *C*^Exp^_Phe_, experimental values of *C*_Phe_; *C*^Pre^_Glc_, predicted values of *C*_Glc_ obtained by the developed ANN model; *C*^Pre^_Phe_, predicted values of *C*_Phe_ obtained by the developed ANN model. The experimental values of *C*_Glc_ and *C*_Phe_ are presented as the means ± SD (n = 3) in Fig. 5

To verify the effectiveness of the proposed ANN model, a testing dataset with suitable ranges of input/output variables was also used to predict *C*_Glc_ and *C*_Phe_ under different dilute inorganic acid pretreatment conditions. The fitting relationships between the predicted and experimental values of *C*_Glc_ and *C*_Phe_ are plotted in Fig. 5. The slope of the fitting curve and the correlation coefficient of *R*^2^ value are the two key parameters for accurately evaluation of the proposed ANN model. In other words, a better fitting relationship between predicted values obtained from the developed model and experimental values generally featured with the slope and *R*^2^ nearing to 1.0. As shown in Fig. 5, the diagonal of the plot (*Y* = *X*) was displayed with a slope of 1.0 for clearly comparison. It is indicated that the *R*^2^ of *C*_Glc_ fitting curve was 0.906 under the range of 7.5–25 g/L, with a slope of 0.86. In addition, the experimental values of *C*_Phe_ were located at 0.8–3.0 g/L. The ANN model obtained the fitting curve with a slope of 0.82 and a *R*^2^ of 0.904 for prediction of *C*_Phe_. Based on the fitting performance shown in Fig. 5, it is concluded that ANN modeling is an efficient tool for predicting *C*_Glc_ and *C*_Phe_ simultaneously from the non-linearity and complexity of the input–output system containing corn stover pretreatment/enzymatic processes.

### Relative importance of input parameters on output variables

For the pretreatment of lignocellulosic biomass, elucidating and understanding the influence of pretreatment or enzymatic conditions on glucose hydrolysis yield and inhibitors formation are beneficial for guiding the biorefinery process (Bhatia et al. 2020). Although numerous studies have investigated the effects of pretreatment and enzymatic methods on glucose yield and inhibitors formation (Hassan et al. 2018; Jönsson and Martín 2016), the quantitative relationship properties between operation variables and those derived contents in biomass hydrolysate before fermentation are still unclear.

**Fig. 6:**
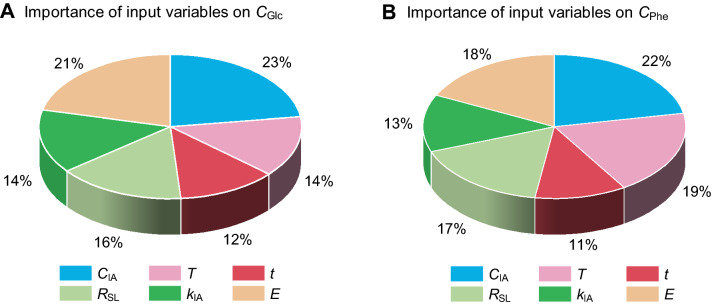
Relative importance of six input parameters on *C*_Glc_ (**A**) and *C*_Phe_ (**B**), which calculated by Eq. (4) with the detailed weights of the developed ANN model shown in Table 3

Focusing on this concern, the relative importance (*I*_i_, *i* = 1, 2…6) of the six input variables on the two output variables of *C*_Glc_ and *C*_Phe_ were analyzed (Fig. 6). The *I*_i_ was calculated by the Garson equation Eq. (4) with the weight matrixes of *IW*_j,i_ and *LW*_k,j_ in the developed ANN model, which are listed in Table 3. As shown in Fig. 6A, the six input variables strongly influenced *C*_Glc_ with the range of 12–23%. The five parameters included in the pretreatment process (*C*_IA_, *T*, *t*, *R*_SL_, and *k*_IA_) represent up to 79% importance, and enzyme dosage (*E*) accounts for 21% importance on *C*_Glc_. Interestingly, the highest importance (*I*_1_ = 23%) on *C*_Glc_ is the concentration of inorganic acid (*C*_IA_), which is even higher than that of enzyme dosage (21%). The results revealed that an efficient pretreatment strategy is beneficial for glucose hydrolysis, which is mainly attributed to the improved accessibility of cellulose during pretreatment process (Siqueira et al. 2017; Xu et al. 2016). The relative importance of *T*, *R*_SL_, and *k*_IA_ on *C*_Glc_ is around 14%-16%, while in this case, the *I*_3_ (*t*) is the lowest index (Fig. 6A). In addition, the relative importance of input variables on *C*_Phe_ is plotted in Fig. 6B. Similarly, inorganic acid concentration (*C*_IA_) also has the strongest importance of 22% on *C*_Phe_. It is indicated that a severe acidic environment with a higher concentration of H^+^ could improve the efficiency of lignin deconstruction coupling with phenolics formation (He et al. 2020). The other four variables in the pretreatment process occupied 60% importance on *C*_Phe_ (i.e*., T* for 19%, *t* for 11%, *R*_SL_ for 17%, and *k*_IA_ for 13%, Fig. 6B). It should be noted that enzyme dosage (*E*) still kept higher importance of 18% on *C*_Phe_ (Fig. 6B), with a relatively lower importance of 21% on *C*_Glc_ (Fig. 6A). The obtained results of the relative importance of enzyme dosage on *C*_Phe_ reflected that the derived phenolic contents would change in biomass hydrolysate during enzymatic hydrolysis.

## Discussion

Optimization of biorefinery process can lead to highly efficient production of biofuels from renewable resources such as lignocellulosic biomass. Traditional optimization method called “*one variable at time*” (OVAT) is time-consuming and requires a large number of experiments. To circumvent the limitation, experimental models are used to elucidate the relationship between operating parameters and final outcomes. Recently, various models such as response surface methodology (RSM) and ANN were reported (Das et al. 2015; Fernandes et al. 2020; Sewsynker-Sukai and Gueguim Kana 2018). The obstacle of RSM is the limitation to the hypothesis of quadratic correlation between conditions because it assumes the second-order polynomial equation (Fernandes et al. 2020). Therefore, ANN modeling was used to build efficient models to predict *C*_Phe_ and *C*_Glc_.

Some ANN models were recently developed to assess the biomass pretreatment for the production of biofuels and fine chemicals (Moodley et al. 2019; Sunphorka et al. 2017; Vani et al. 2015). Compared with those models, the proposed ANN model in this study possessed three advantages. Firstly, the derived feature of the diversity of phenolic compound content from dilute inorganic acid pretreatment of lignocellulosic biomass, which is one of the most promising approaches to industrial implementation, was clarified when using corn stover as the feedstock for the first time. Secondly, the dilute inorganic acid pretreatment and enzymatic hydrolysis processes were considered from a systematic perspective. It is because *C*_Phe_ and *C*_Glc_ in biomass hydrolysate are the key factors affecting the overall fermentation performance. Lastly, the relationship of relative importance between operation variables and output variables (*C*_Phe_ and *C*_Glc_) was clearly elucidated by the weight matrixes in the developed ANN model. It should be noted that although ANN models could not accurately calculate/predict results beyond the range of operational parameters in training/validation datasets, it can still provide an estimation of parameters in an uncharted workspace by catching the trends during the training process (Rashid et al. 2021). Collection of previously reported data for the development of advanced ANN models would be an efficient strategy. Therefore, it is concluded that ANN modeling is a powerful tool for predicting key parameters in some crucial multivariate non-linear bioprocesses. The relative importance analysis also provides new insights into the biochemical process assessment and optimization.

Although the phenolic compounds were directly formed by lignin degradation during pretreatment process, some studies found that the content (*C*_Phe_) still changes during the enzymatic hydrolysis process. It is mainly attributed to the interactions effect between cellulase and lignin-derived phenolics (Yao et al. 2021; Zhao et al. 2021). In addition, the water-soluble lignin-derived phenolics were adsorbed by cellulase and inhibit the enzymatic efficiency (Yuan et al. 2021). Those findings could explain the result that the enzyme dosage has a high importance of 18% on *C*_Phe_ (Fig. 6B).

Although dilute inorganic acid pretreatment was selected to elucidate the derived feature of phenolic content from corn stover, some other indispensable factors should also be considered to further improve the sustainability of biorefinery process. Different pretreatment methods have various solubilization abilities of lignin, cellulose, and hemicellulose. For the removal efficiency of lignin, the alkaline-based hydrolysis is generally higher than that of dilute acid-based methods (Zabed et al. 2016). If the aim is only to investigate lignin removal efficiency from lignocellulosic biomass, alkaline-based pretreatment might be a better choice. In addition, the operation parameters including the particle size and biomass species also influenced the fermentable sugars and derived phenolic contents (Vani et al. 2015), which should be explored in the future.

## Conclusions

An artificial neural network (ANN) model was developed to simultaneously predict the derived feature of phenolic compounds content (*C*_Phe_) and glucose yield (*C*_Glc_) in biomass hydrolysate from dilute inorganic acid pretreatment and enzymatic hydrolysis. Five pretreatment and enzyme dosage parameters were used as the input variables in the optimized ANN model, which has one hidden layer with 12 neurons. Results indicated that the developed ANN model has a good fitting performance (*R*^2^ > 0.90) for the prediction of *C*_Phe_ and *C*_Glc_. The relative importance of six variables on *C*_Phe_ and *C*_Glc_ was also calculated to provide new insights for optimizing biorefinery to produce biofuels.

### Supplementary Information

**Additional file 1: Table S1**. The input data for development of ANN model to predict *C*_Glc_ and *C*_Phe_ in biomass hydrolysate after dilute inorganic acid pretreatment and enzymatic hydrolysis.

## Data Availability

The data supporting the conclusions of this article are included in the main manuscript.
