# Supplementary material for: Prediction of phenolic compounds and glucose content from dilute inorganic acid pretreatment of lignocellulosic biomass using artificial neural network modeling
Source: Bioresour Bioprocess. 2021 Dec 19;8(1):134. doi: 10.1186/s40643-021-00488-x (PMC10992208; doi:10.1186/s40643-021-00488-x)
Supplement: Supplementary file 1 — Additional file 1: Table S1. The input data for development of ANN model to predict CGlc and CPhe in biomass hydrolysate after dilute inorganic acid pretreatment and enzymatic hydrolysis. [file 40643_2021_488_MOESM1_ESM.docx]

**Table S1.** The input data for development of ANN model to predict *C*_Glc_ and *C*_Phe_ in biomass hydrolysate after dilute inorganic acid pretreatment and enzymatic hydrolysis.

| Samples | The six processing parameters as the input variables in ANN model *^a^* | | | | | | Output variables *^b^* | |
| --- | --- | --- | --- | --- | --- | --- | --- | --- |
|  | *C*_IA_ (mol/L) | *T* (°C) | *T* (min) | *R*_SL_ | *k*_IA_ | *E* (FPU/g) | *C*_Glc_ (g/L) | *C*_Phe_ (g/L) |
| 1 | 0.05 | 160 | 60 | 0.10 | 1 | 20 | 22.8 ± 0.85 | 1.134 ± 0.056 |
| 2 | 0.6 | 120 | 20 | 0.10 | 2 | 20 | 3.50 ± 0.13 | 1.100 ± 0.012 |
| 3 | 0.05 | 200 | 60 | 0.10 | 1 | 10 | 8.50 ± 0.21 | 1.186 ± 0.030 |
| 4 | 0.1 | 200 | 20 | 0.10 | 2 | 20 | 15.00 ± 0.43 | 2.481 ± 0.100 |
| 5 | 0.1 | 120 | 60 | 0.10 | 1 | 20 | 26.40 ± 1.21 | 1.245 ± 0.043 |
| 6 | 0.6 | 180 | 60 | 0.10 | 3 | 20 | 23.40 ± 0.65 | 1.329 ± 0.045 |
| 7 | 0.1 | 140 | 60 | 0.10 | 1 | 20 | 25.80 ± 1.32 | 1.358 ± 0.056 |
| 8 | 0.2 | 120 | 20 | 0.10 | 2 | 20 | 28.20 ± 0.68 | 0.796 ± 0.067 |
| 9 | 0.05 | 140 | 20 | 0.10 | 2 | 20 | 18.90 ± 0.65 | 1.406 ± 0.010 |
| 10 | 0.2 | 140 | 60 | 0.10 | 3 | 20 | 23.40 ± 1.21 | 0.529 ± 0.003 |
| 11 | 0.2 | 160 | 60 | 0.10 | 3 | 20 | 24.60 ± 0.98 | 0.573 ± 0.004 |
| 12 | 0.2 | 120 | 60 | 0.10 | 1 | 20 | 24.30 ± 1.54 | 1.274 ± 0.006 |
| 13 | 0.4 | 140 | 60 | 0.10 | 1 | 20 | 23.60 ± 0.21 | 0.777 ± 0.004 |
| 14 | 0.4 | 180 | 60 | 0.10 | 1 | 20 | 2.90 ± 0.43 | 0.980 ± 0.007 |
| 15 | 0.1 | 120 | 60 | 0.10 | 2 | 20 | 21.00 ± 0.54 | 1.089 ± 0.054 |
| 16 | 0.4 | 200 | 60 | 0.10 | 1 | 20 | 1.60 ± 0.01 | 2.259 ± 0.130 |
| 17 | 0.6 | 120 | 60 | 0.10 | 1 | 20 | 20.46 ± 0.69 | 1.619 ± 0.110 |
| 18 | 0.2 | 180 | 60 | 0.15 | 2 | 20 | 8.20 ± 0.03 | 2.529 ± 0.160 |
| 19 | 0.6 | 140 | 60 | 0.10 | 1 | 20 | 22.33 ± 0.56 | 1.879 ± 0.100 |
| 20 | 0.6 | 160 | 60 | 0.10 | 1 | 10 | 8.14 ± 0.32 | 2.767 ± 0.120 |
| 21 | 0.4 | 160 | 60 | 0.10 | 3 | 20 | 23.40 ± 1.20 | 0.616 ± 0.054 |
| 22 | 0.1 | 200 | 60 | 0.10 | 2 | 20 | 6.80 ± 0.43 | 2.006 ± 0.210 |
| 23 | 0.6 | 200 | 60 | 0.10 | 1 | 20 | 2.64 ± 0.02 | 2.397 ± 0.043 |
| 24 | 0.2 | 200 | 60 | 0.10 | 1 | 20 | 1.80 ± 0.01 | 2.370 ± 0.024 |
| 25 | 0.05 | 120 | 20 | 0.10 | 2 | 20 | 15.20 ± 0.24 | 0.750 ± 0.043 |
| 26 | 0.2 | 140 | 60 | 0.10 | 1 | 20 | 27.80 ± 1.26 | 1.575 ± 0.012 |
| 27 | 0.6 | 140 | 60 | 0.10 | 3 | 20 | 25.00 ± 0.64 | 1.419 ± 0.056 |
| 28 | 0.05 | 180 | 20 | 0.15 | 2 | 15 | 28.00 ± 0.43 | 1.327 ± 0.098 |
| 29 | 0.05 | 120 | 60 | 0.10 | 2 | 20 | 20.20 ± 0.70 | 1.110 ± 0.032 |
| 30 | 0.1 | 120 | 20 | 0.10 | 2 | 20 | 24.00 ± 1.67 | 0.785 ± 0.065 |
| 31 | 0.2 | 180 | 60 | 0.10 | 3 | 20 | 23.60 ± 1.32 | 0.626 ± 0.012 |
| 32 | 0.1 | 140 | 20 | 0.10 | 2 | 20 | 26.40 ± 0.43 | 1.168 ± 0.008 |
| 33 | 0.6 | 160 | 60 | 0.10 | 3 | 20 | 24.40 ± 0.44 | 1.345 ± 0.006 |
| 34 | 0.1 | 160 | 20 | 0.10 | 2 | 20 | 25.40 ± 0.14 | 1.097 ± 0.003 |
| 35 | 0.1 | 180 | 20 | 0.10 | 2 | 20 | 23.00 ± 0.30 | 1.385 ± 0.012 |
| 36 | 0.2 | 200 | 60 | 0.15 | 2 | 20 | 3.00 ± 0.02 | 2.619 ± 0.087 |
| 37 | 0.6 | 200 | 20 | 0.10 | 2 | 20 | 4.80 ± 0.12 | 2.875 ± 0.120 |
| 38 | 0.05 | 140 | 60 | 0.10 | 1 | 20 | 15.90 ± 0.21 | 0.967 ± 0.103 |
| 39 | 0.4 | 180 | 60 | 0.10 | 3 | 20 | 24.60 ± 0.80 | 0.655 ± 0.032 |
| 40 | 0.2 | 160 | 20 | 0.10 | 2 | 20 | 13.80 ± 0.43 | 1.157 ± 0.110 |
| 41 | 0.2 | 180 | 20 | 0.10 | 2 | 20 | 10.40 ± 0.21 | 1.667 ± 0.122 |
| 42 | 0.2 | 200 | 20 | 0.10 | 2 | 20 | 4.20 ± 0.20 | 1.237 ± 0.032 |
| 43 | 0.4 | 120 | 20 | 0.10 | 2 | 20 | 19.50 ± 0.23 | 0.809 ± 0.076 |
| 44 | 0.4 | 140 | 20 | 0.10 | 2 | 20 | 13.20 ± 0.54 | 3.010 ± 0.020 |
| 45 | 0.4 | 140 | 60 | 0.10 | 3 | 20 | 18.60 ± 0.86 | 0.573 ± 0.032 |
| 46 | 0.4 | 160 | 20 | 0.10 | 2 | 20 | 9.20 ± 0.12 | 0.914 ± 0.012 |
| 47 | 0.4 | 200 | 20 | 0.10 | 2 | 20 | 3.80 ± 0.20 | 1.744 ± 0.032 |
| 48 | 0.6 | 160 | 20 | 0.10 | 2 | 20 | 4.60 ± 0.33 | 1.332 ± 0.043 |
| 49 | 0.4 | 160 | 60 | 0.15 | 2 | 15 | 19.40 ± 0.65 | 1.913 ± 0.105 |
| 50 | 0.4 | 180 | 60 | 0.10 | 2 | 20 | 3.80 ± 0.10 | 2.378 ± 0.121 |
| 51 | 0.2 | 140 | 20 | 0.10 | 3 | 20 | 26.00 ± 0.43 | 1.371 ± 0.056 |
| 52 | 0.2 | 160 | 20 | 0.10 | 3 | 20 | 28.60 ± 1.32 | 1.583 ± 0.089 |
| 53 | 0.4 | 140 | 20 | 0.10 | 3 | 20 | 23.20 ± 1.03 | 1.216 ± 0.102 |
| 54 | 0.4 | 160 | 20 | 0.10 | 3 | 20 | 28.30 ± 1.06 | 1.784 ± 0.027 |
| 55 | 0.4 | 180 | 20 | 0.10 | 3 | 20 | 20.40 ± 0.90 | 1.496 ± 0.007 |
| 56 | 0.2 | 160 | 20 | 0.10 | 1 | 20 | 25.20 ± 0.87 | 1.028 ± 0.010 |
| 57 | 0.2 | 140 | 20 | 0.10 | 1 | 20 | 25.20 ± 0.65 | 1.586 ± 0.014 |

*Note:*

The experimental results of *C*_Phe_ and *C*_Glc_ for development of ANN model are represented as the mean ± standard deviation (SD) of three independent experiments.

*^a^*: *C*_IA_, inorganic acid concentration (mol/L); *T*, pretreatment temperature (°C); *t*, residence time (min), *R*_SL_, solid to liquid ratio; *k*_IA_, kinds of inorganic acids (“1” for HCl, “2” for H_2_SO_4_, and “3” for H_3_PO_4_); *E*, enzyme loading dosage (FPU/g corn stover).

*^b^: C*_Glc_, glucose concentration in biomass hydrolysate after pretreatment and enzymatic hydrolysis (72 h); *C*_Phe_, phenolic content in biomass hydrolysate after pretreatment and enzymatic hydrolysis (72 h).
